# Cellular immune response of *Staphylococcus aureus* enterotoxin B in Balb/c mice through intranasal infection

**DOI:** 10.14202/vetworld.2022.1765-1771

**Published:** 2022-07-24

**Authors:** Hidayatun Nisa Purwanasari, Amanda Tri Utami Permatasari, Fajar Budi Lestari, Madarina Wasissa, Khusnan Zaini, Siti Isrina Oktavia Salasia

**Affiliations:** 1 Department of Clinical Pathology, Faculty of Veterinary Medicine, Universitas Gadjah Mada, Yogyakarta, Indonesia; 2 Department of Bioresources Technology and Veterinary, Vocational College, Universitas Gadjah Mada, Yogyakarta, Indonesia; 3 Interdisciplinary Program of Biomedical Sciences, Faculty of Graduate School, Chulalongkorn University, Bangkok, Thailand; 4 Academy of Farming Brahmaputra, Yogyakarta, Indonesia

**Keywords:** enterotoxin B, hematology, histopathology, intranasal, *Staphylococcus aureus*

## Abstract

**Background and Aim:**

*Staphylococcus aureus* produces various superantigen exotoxins, including staphylococcal enterotoxin B (SEB). It causes fatal anaphylactic reactions and toxic shock. This study aimed to evaluate the reaction of leukocytes and histopathological changes in the respiratory organs of Balb/c mice after intranasal infection with enterotoxigenic *S. aureus* (SEB).

**Materials and Methods:**

The presence of the *seb* gene in *S. aureus* was established in this study using polymerase chain reaction-specific primer. Two groups of 8-week-old male Balb-c mice consist of six mice in each group. The treated group was infected with 50 μL and 100 μL of SEB intranasal on days 1 and 14, respectively. NaCl was administered in the second group and was considered as a control group. Blood samples were collected through the retro-orbital plexus on days 1, 4, 7, 14, and 22 after infections. Total cell counts were analyzed with an independent sample t-test and compared using the statistical package for the social sciences (SPSS) version 16.0 (IBM Corp., NY, USA). The infected tissues of the respiratory organ were observed descriptively and compared to the control group.

**Results:**

The *seb* gene with a molecular size of 478 bp, indicating the SEB strain, is present in *S. aureus* used in this study. Intranasal administration of SEB showed increased leukocytes, lymphocytes, monocytes, and eosinophils on day 22 post-infection. Significant leukocytosis was seen on days 6 and 14; lymphocytosis on days 1, 4, 6, and 16; and eosinophilia on days 6, 14, and 22 compared with the control group (p > 0.05). In contrast, the neutrophil decreased after an increase of immature band cells compared to the control group, indicating a severe acute infection with SEB. The lungs and trachea of the test group had an inflammatory cell accumulation in the respiratory organ.

**Conclusion:**

Intranasal route infection of *S. aureus* containing *seb* gene significantly induced the cellular immune response and caused pathological changes in the respiratory tissues of the Balb/c mice model. The hematological changes were aligned with marked pathological changes in the respiratory tract. Balb/c mice could be an excellent experimental model to study toxic and anaphylactic shock against SEB to define the future therapeutic agents.

## Introduction

*Staphylococcus aureus* frequently colonizes the skin and upper airways. It can be pathogenic in several chronic airway disorders [1]. An increased colonization rate of *S. aureus* in nasal polyp tissue has been reported [2]. *S. aureus* can manipulate host immune responses by producing superantigens that facilitate invasion and colonization [3]. *S. aureus* produces various superantigen exotoxins (SAEs). Enterotoxins cause a toxic shock-like syndrome, food poisoning, and some allergic and autoimmune diseases [4]. Staphylococcal enterotoxins (SEs) acting as superantigens can induce an intense T-cell activation by releasing Type 2 cytokines. Interleukin (IL)-4, IL-5, and IL-13 by acting on Th2 cells, can promote a polyclonal IgE response and eosinophilic inflammation [1, 5]. About 24 different enterotoxins and related toxins have been described in *S. aureus* with some differences in structure and biological activity [6]. Staphylococcal enterotoxin B (SEB) is the most potent SE since it can cause multiple organ system failure and death at even low concentrations. This toxin is produced in large quantities by *S. aureus* and is possibly the main responsible for the pathological conditions induced by this bacterium [7]. The ability of SEB stimulates systemic immune activation after exposure through non-enteric mucous membranes, primarily through the nasal tract. The nasal passage is the most common site of staphylococcal colonization. Since *S. aureus* in the carrier state can elaborate various superantigens, there is a possibility that the nasal passage may be exposed to SEB [8].

Animal models have great potential as powerful tools to help answer some of the difficult questions as human research is limited by ethical concerns and the possibility of fatal anaphylactic reactions [9]. *In vivo* animal models are important to develop therapeutics against SEB-induced anaphylactic toxic shock. At present, the SEB shock model requires pretreatment with various agents to increase the sensitivity of mice to SEB [10]. However, these models are less than ideal, relying on artificial conditions and manipulation of the immune response. Researchers need an excellent experimental model to address the development of a vaccine or therapy against the SEB agent. In this study, Balb/c mice were intranasally induced with *S. aureus* strain containing enterotoxin B to obtain a natural immune response that could be an excellent experimental model to study toxic and anaphylactic shock against SEB to determine the future therapeutic agents. *S. aureus*, which contains SEB, was chosen as an inflammatory agent in this study because SEB is one of the SAE commonly detected in the nasal area and has been linked to allergic diseases such as nasal polyps or asthma. The pathogenesis of SEB that enters the intranasal route remains unclear. Intranasal exposure to SEB is possibly due to the colonization of *S. aureus* [1, 8].

This study aimed to evaluate the cellular immune response of Balb/c mice, including leukocyte response and histopathological changes of respiratory organs after being infected with enterotoxigenic *S. aureus* (SEB) intranasally.

## Materials and Methods

### Ethical approval

All procedures performed in this research were approved by the Animal Care and Use Committee, Faculty of Veterinary Medicine, Universitas Gadjah Mada (No. 00114/EC-FKH/Int./2021).

### Study period and location

This study was conducted from June to September 2021 at the Clinical Pathology Laboratory, Faculty of Veterinary Medicine, Universitas Gadjah Mada.

### Identification of SEB

The SEB human strain was obtained from the Regional Health Laboratory, Yogyakarta. *S. aureus* containing *seb* gene was confirmed using polymerase chain reaction (PCR)-specific primer. The strains have been identified as *S. aureus* based on phenotypic and genotypic identification at the Laboratory of Clinical Pathology, Faculty of Veterinary Medicine, Universitas Gadjah Mada. Phenotypic identifications included mannitol salt agar, coagulase, Gram staining, and catalase [11]. Molecular identifications were performed by detecting the 23S rRNA and *nuc* genes, as described by Windria *et al*. [12]. The detection of enterotoxin encoding *seb* gene was carried out using multiplex PCR method with the primers, F: TCTGAACCTTCCCATCAAAAAC and R: TCGCATCAAACTGACAAACG, and the programs used were as follows: 35 cycles at 95°C for 15 min, 95°C for 30 s, 57°C for 90 s, 72°C for 90 s, and 72°C for 10 min.

The PCR products were analyzed by electrophoresis in 1.5% agarose gel (Invitrogen, USA) stained by SYBR Safe (Invitrogen) in 1× TBE (Tris base, boric acid, and ethylenediaminetetraacetic acid [EDTA]) buffer. The resulting bands were visualized on a UV transilluminator.

### SEB suspension

The molecularly confirmed SEB was subsequently cultured on blood agar (Oxoid, Germany) for 24 h at 37°C. A colony from blood agar was recultured in Todd-Hewitt broth for 24 h at 37°C. The broth with bacterial growth was centrifuged at 1000× *g* for 15 min. Distilled water was added to the bacterial pellet after removing the supernatant until its turbidity resembled a 0.5 McFarland solution (1.5 × 10^8^ colony-forming units/mL).

### Intranasal SEB challenge to animal models

Twelve 8-week-old male Balb/c mice, 20–40 g in weight, were obtained from Integrated Research and Testing Laboratory (LPPT), Universitas Gadjah Mada. Mice were divided into two groups (control and SEB test groups) to evaluate the immunogenicity. Mice in the control group received NaCl 50 μL intranasally (i.n.) with a micropipette. Mice in the SEB test group were intranasally administered with 50 μL and 100 μL of the bacterial suspension on days 1 and 14, respectively. Before i.n. administration, the mice were previously anesthetized intraperitoneal (i.p.) with the combination of ketamine and xylazine.

### Hematological examinations

Before collecting blood, mice were anesthetized (i.p.) with a combination of ketamine and xylazine solution. Blood samples were collected on days 1, 4, 7, 14, and 22 through the retro-orbital plexus with an EDTA anticoagulant. Blood samples were analyzed using a veterinary hematology analyzer (Hematology Analyzer Mindray BC 2800 Vet, Shenzhen Mindray Bio-Medical Electronics Co. Ltd., Shenzhen, China). Differential leukocyte counts were manually calculated from a thin blood smear under a light microscope (Olympus, Tokyo, Japan).

### Histopathological examinations

The mice were euthanized using lethal doses of an anesthetic agent on day 22. The trachea and lungs were collected during necropsy. The selected organs were fixed in 10% formalin solution and were processed for standard histological preparation using routine hematoxylin and eosin staining. The histopathological changes in the tissues were then analyzed using a light microscope (Olympus).

### Statistical analysis

Hematology data were calculated as mean ± standard deviation. Total blood cell counts were analyzed using an independent sample t-test and compared using SPSS version 16.0 (IBM, Armonk, USA). The significant difference was placed at p < 0.05. The infected tissues of the respiratory organ were descriptively observed and compared with the control group.

## Results

### SEB

**Figure-1:**
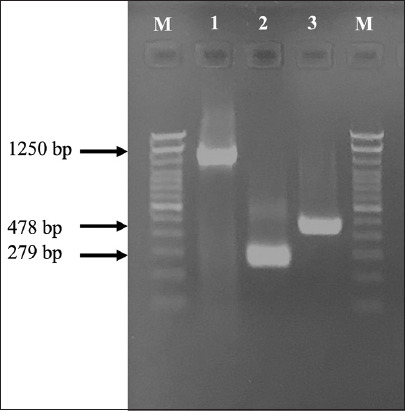
Amplicon of the 23S rRNA (1250 bp), *nuc* (279 bp), and *seb* gene of *Staphylococcus aureus* (478 bp) and M = Marker 100 bp molecular size DNA ladder.

According to the results of biochemical characteristics and amplification of the 23S rRNA in 1250 bp, *nuc* in 279 bp, and specific for *S. aureus*, the isolate used in this study was identified as *S. aureus*. The enterotoxin encoding *seb* gene has a molecular size of around 478 bp (Figure-1).

### Hematological examinations

**Figure-2:**
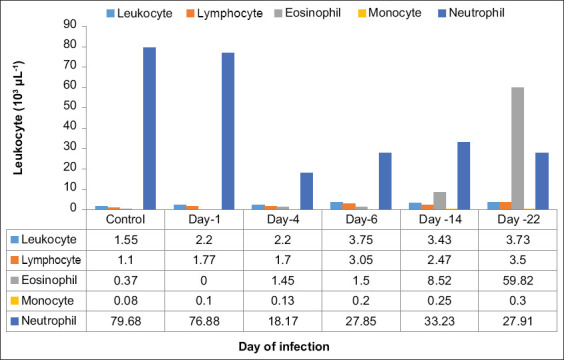
Comparison of leukocytes of mice after being induced by staphylococcal enterotoxin B from day 1 until day 22.

Hematology examination results of both groups were compared with the mean of all blood parameters, as summarized in Table-1. Figure-2 shows the calculated total of leukocytes, lymphocytes, and eosinophils from days 1 to 22. Data comparisons were also analyzed using SPSS (independent sample t-test). Intranasal administration of SEB revealed increased leukocytes, lymphocytes, monocytes, and eosinophils on day 22 after infection.

**Table 1:** Comparison of the mean blood parameters of mice after being induced with staphylococcal enterotoxin B.

Cells	Control (10^3^ µL^−1^)	Day-1 (10^3^ µL^−1^)	Day-4 (10^3^ µL^−1^)	Day-6 (10^3^ µL^−1^)	Day-14 (10^3^ µL^−1^)	Day-22 (10^3^ µL^−1^)
Leukocyte	1.55	2.20	2.20	3.75	3.43	3.73
Lymphocyte	1.10	1.77	1.70	3.05	2.47	3.50
Eosinophil	0.37	0	1.45	1.50	8.52	59.82
Monocyte	0.08	0.10	0.13	0.20	0.25	0.30
Neutrophil	79.68	76.88	18.17^b^	27.85^b^	33.23^b^	27.91^b^

b Band neutrophils observed

The leukocyte counts in mice induced by SEB were significantly elevated on days 6 and 14, compared to the control group (p > 0.05), followed by neutropenia with a left shift. Leukocyte counts began to increase on 6 days post-infection (3.75 × 10^3^ μL^−1^), slightly decreased on 14 days (3.43 × 10^3^ μL^−1^), and after second i.n. SEB infection increased on day 22 (3.73 × 10^3^ μL^−1^). On days 6, 14, and 22, absolute eosinophil counts were significantly elevated (p > 0.05). Eosinophils gradually increased on 4 days after infection (1.45 × 10^3^ μL^−1^) and on 6 days (1.50 × 10^3^ μL^−1^), markedly increased on 14 days (8.52 × 10^3^ μL^−1^), and, after second i.n. SEB infection on day 22, surprisingly jumped to 59.82 × 10^3^ μL^−1^, indicating severe anaphylactic shock. The total lymphocyte counts were significantly increased on days 1, 4, 6, and 14, compared with the control group (p > 0.5). Lymphocytes began to increase 1 day after infection (1.77 × 10^3^ μL^−1^), peaked on 6 days (3.05 × 10^3^ μL^−1^), and decreased on 14 days (2.47 × 10^3^ μL^−1^) and after the second i.n. SEB infection increased on day 22 (3.50 × 10^3^ μL^−1^). There were no significant differences in monocyte counts between the investigated mice. Table-2 shows a comparison of statistical values for the total count of leukocytes, lymphocytes, and eosinophils.

**Table 2:** p-values obtained from the statistical package for the social sciences.

Cells	Control (10^3^ µL^−1^)	Day-1 (10^3^ µL^−1^)	Day-4 (10^3^ µL^−1^)	Day-6 (10^3^ µL^−1^)	Day-14 (10^3^ µL^−1^)	Day-22 (10^3^ µL^−1^)
Leukocyte	0.440	0.051	0.054	0.007*	0.000*	0.058
Lymphocyte	0.132	0.005*	0.015*	0.007*	0.001*	0.074
Eosinophil	0.388	0.363	0.11	0.002*	0.004*	0.006*

* Significant difference (p < 0.05)

### Histopathological examinations

**Figure-3:**
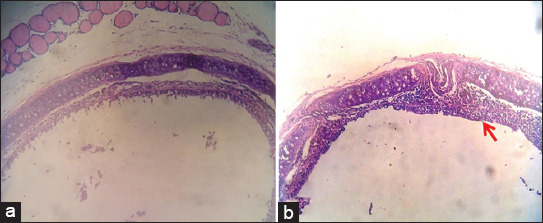
The photomicrograph of mice trachea from the (a) negative control group compared to (b) staphylococcal enterotoxin B-infected mice that markedly showed pathological changes with inflammatory cells accumulation (arrow) covering alongside of tracheal surface epithelial (H&E, ×100).

**Figure-4:**
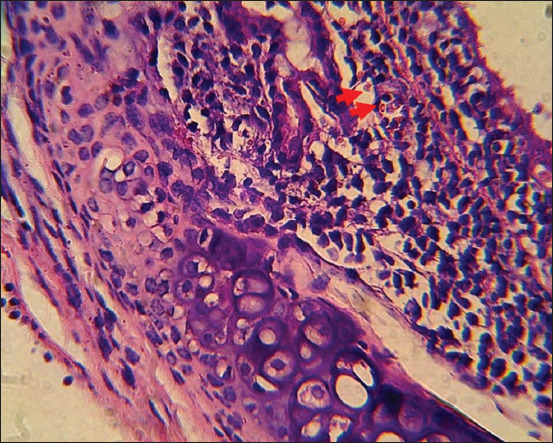
Detail magnification of mice trachea infected by staphylococcal enterotoxin B showing the infiltration of polymorphonuclear and mononuclear inflammatory cells, eosinophil (arrow) (H&E, ×400).

Marked histopathological changes were found along the respiratory tract of SEB-treated mice. An abundant accumulation of inflammatory cells was observed on the tracheal mucosal surface (Figure-3). The pathological change was consistent with the previous studies that mentioned that SEB infection causes mucosal respiratory tract damage [13]. Detailed changes are shown in Figure-4 with the accumulation of polymorphonuclear and mononuclear inflammatory cells. Eosinophils were found within the accumulation.

**Figure-5:**
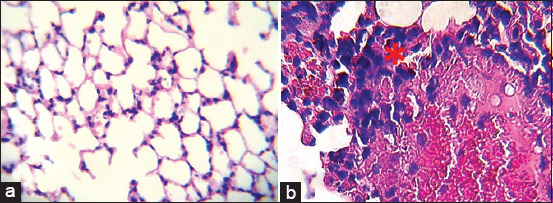
The photomicrograph of (a) negative control mice lung compared to (b) staphylococcal enterotoxin B-infected mice showed the marked changes in thickening of interstitial alveolar-septa containing inflammatory cells that predominantly mononuclear cells (*). Edema fluid and inflammatory cells within alveoli were also found (H&E, ×400).

Histopathological changes were also observed in the lower respiratory tract. Atelectasis characterized by thickening of the interstitial alveolar septa of lungs with inflammatory cell infiltration complicated by alveoli filled with edema fluid was also found (Figure-5).

## Discussion

*S. aureus* used in this study contained the *seb* gene with a molecular size of about 478 bp, indicating a SEB strain. This SEB strain was isolated from humans in Yogyakarta, Central Java, Indonesia. *S. aureus* containing the gene encoding SEs A, B, C, G, H, and I have been isolated from the milk of dairy cows and goats [11, 14]. Enterotoxins A, B, E, G, H, and I were found in food products, cattle, and humans [15], in Central Java, Indonesia, indicating the potential for the spread of enterotoxins harmful to public health.

SEB is one of the enterotoxin proteins and is responsible for several extensive pathophysiological changes in humans and mammals and triggers an excessive cellular immune response leading to toxic shock. SEBs comprise a large group of proteins and are 19–29 kDa polypeptides in the bacterial SA protein family [6]. SEB can activate the Toll-like receptor 2 (TLR2). TLR is essential in recognizing bacterial components to induce an appropriate immune response against the microorganism encountered. Bacterial SAs are a family of potent immunostimulatory exotoxins that activate T-lymphocytes. The T cell receptor (TCR) molecule and the Type II major histocompatibility complex (MHC-II) are two natural receptors that bind to staphylococcal SA. SEB can ligate with the β-chain of TCR to induce hyperinflammatory responses and autoimmune reactions [10].

An increased presence of *S. aureus* enterotoxins on respiratory mucosa is linked to several diseases, including asthma, nasal polyp, and allergic rhinitis [1]. *S. aureus* colonization of the nasal mucosa can facilitate its invasion into the subepithelial regions where *S. aureus* secretes proteins that act as superantigens to activate T and B cells [13]. Bacterial superantigens are produced by *S. aureus* and *Streptococcus pyogenes*, which are ubiquitous in nature and can cause severe hemodynamic shock and multiorgan failure. Humans are natural carriers of these organisms, with the nasal passage being the most common site for *S. aureus* colonization. While most pathogenic isolates of *S. aureus* produce one or more enterotoxins, even strains isolated from asymptomatic carriers can produce superantigens [5, 16].

Several studies have investigated the effects of SEs on other inflammatory cells such as eosinophils, macrophages, and mast cells, although SEs mainly affect lymphocyte activation. It has been shown that SEs can promote eosinophils’ survival by inhibiting eosinophil apoptosis [17]. In addition, SEs can act on macrophages by inducing the production of cytokines such as IL-8 and IL-12 and neutrophilic chemotactic factors [1]. *S. aureus* protein A can induce cross-linking of IgE molecules in mast cells, which increases the release of histamine, tryptase, and leukotriene C4 [18].

*S. aureus* containing *seb* gene encoding SEB used in this study changed the cellular immune response with increase in leukocytes, lymphocytes, monocyte, and eosinophils on day 6 post-infection (Table-1). The total leukocytes of the SEB-induced mice were significantly increased compared with the control group on days 6 and 14 (p < 0.05) followed by neutropenia with a left shift. Intranasal administration of SEB caused a significant increase in leukocytes in all the mice investigated (Figure-2). Leukocyte changes and their differences are used as an indicator of the body’s immune system response to pathogens. The increase in total leukocytes correlates with an increase in the number of cells produced by the bone marrow and their migration from the circulation to the tissues [19]. SEBs have the ability to cause systemic immune activation after exposure through the nasal tract [8]. Neutropenia with a left shift indicated the severity of acute SEB infection. Neutrophils are needed to phagocyte the bacteria in the nasal tract. Neutrophils respond to bacterial respiratory tract infections and colonize respiratory tissues. Consequently, neutrophils seem to be reduced in circulation. This condition stimulated the bone marrow to release young neutrophils (band cells), resulting in neutropenia with a left shift [20].

The total eosinophil counts showed a significant increase in eosinophils (eosinophilia) (Figure-1) on days 6, 14, and 22 (p > 0.05), particularly after SEB infection on day 22, indicating severe anaphylactic shock. These data correspond to a previous study by Hellings *et al*. [21]. *S. aureus* is capable of producing 25–30 kDa exotoxin, one of which is SE with the gene *seb*. SEB can increase the potential for allergies, such as skin inflammation, increased eosinophils in models affected by allergic rhinosinusitis, and polypoid nasal lesions [22]. SEB promotes the release of Type 2 cytokines IL-4 and IL-13 by acting on Th2 cells and promoting eosinophilic infiltration [5]. SEB also mediated eosinophil influx because higher IL-5 level leads to enhanced eosinopoiesis and bronchial influx of eosinophils [21]. The number of eosinophils in the circulating blood increases in the late stages of allergic inflammation and usually remains high compared to the inflammatory cells themselves [23]. Eosinophils are chemotactic cells that migrate from the bone marrow into the bloodstream and end up in inflamed tissues. Therefore, eosinophil chemotaxis is important as a target for anti-allergic drugs. Eosinophil activation through different surface receptors allows the release of mediators such as lipids and cytokines through different mechanisms such as exocytosis, gradual degranulation, and cytolysis [24].

The results of the lymphocyte examination show that the number of lymphocytes increased (lymphocytosis) significantly after induction with SEB compared to the control group starting from day 6. An increase in lymphocytes is needed in bacterial infections to assist in the production of lymphokines that act as chemoattractants to stimulate the emergence of cellular immunity in the body [19]. Monocytes also doubled by day 6 and continued to increase through day 22. Bacterial superantigens can upregulate TLR expression in primary human monocytes by ligation of MHC Class II [25].

The pathogenic mechanism of SEB causing respiratory disorder remains unclear. Therefore, an investigation of the detailed pathological changes is needed to understand its pathogenicity. This study found marked pathological changes in the trachea, particularly along the epithelial surface. The result agrees with the previous study that described complex immune regulations during SEB infection that resulted in nasal epithelial damage [13].

Several pathological changes occurred in the lungs, such as thickening alveolar septa with inflammatory cells, edema fluid, and inflammatory cells within the alveoli. Along the trachea, there was also an accumulation of inflammatory cells. Intranasal inoculation of *S. aureus* containing SEB caused trachea and lung inflammation and systemic immune system activation. In addition, nasal SEB increased the bronchial expression of eotaxin-1, which acts synergistically with IL-5 to recruit eosinophils from the blood to the inflamed airways [26]. Pro-inflammatory cytokines and chemokines, which are inflammatory mediators, can be produced in response to SEs. These inflammatory mediators can cause leukocyte migration and tissue damage. These inflammatory mediators cause a hyperacute release of the cytokines TNF-a, IL-1 and IL-2, IL-10, IFN-g, monocyte chemoattractant protein-1 (MCP-1), and others derived from T cells.

SE has an exotoxin capable of bypassing normal antigen-processing mechanisms and binds to MHC-II molecules on antigen-presenting cells and V regions. SEs can activate and stimulate T-cell formation, which is then referred to as superantigens. The ability of superantigen affinity allows microbes to excrete toxins, disrupt the body’s defense system, increase pro-inflammatory cytokines, chemokines, and lytic enzymes, and activate inflammatory and coagulation processes. T-helper 1 cells work together with TNF and IL-1 to trigger immune reactions and tissue damage. IL-2, as a result of the activation of superantigens by T cells, causes vasodilation and damage to blood vessels leading to edema. Chemokines, MCP-1, and the presence of IL-8 and macrophage inflammatory protein-1a, which are the result of direct induction by SE, cause the migration of leukocytes, neutrophils, and dendritic cells to infected tissues [10, 20].

Cellular immune response studies in the Balb/c mouse model describe the clinicopathological changes caused by enterotoxigenic *S. aureus* infection [27]. Lestari *et al*. [28] have also reported resistance of enterotoxin-containing *S. aureus* to the cellular immune defense system.

## Conclusion

It can be concluded that infection of *S. aureus* containing enterotoxin B, through the intranasal route of Balb/c mice, could significantly induce the cellular immune response, marked by increasing the leukocytes’ lymphocytes, monocytes, and eosinophils and decreasing the neutrophil with immature band cells. Eosinophil findings might correlate to the allergic response due to SEB infection. The pathological changes in the respiratory tract were correlated with the hematological changes. To study toxic and anaphylactic shock against SEB, and determine future therapeutic agents, Balb/c mice could be an excellent experimental model.

## Authors’ Contributions

HNP: Performed the experiment, data analysis, and drafted the manuscript. ATUP: Performed the experiment, data analysis and reviewed the manuscript. FBL: Data analysis and drafted the manuscript. MW: Data analysis and reviewed the manuscript. KZ: Supervised and reviewed the manuscript. SIOS: Conceptualized and supervised the study and drafted the manuscript. All authors have read and approved the final manuscript.
